# Acthar Gel Inhibits the Activation of CD4^+^ and CD8^+^ T Cells

**DOI:** 10.1089/jir.2022.0257

**Published:** 2023-04-12

**Authors:** Dale Wright, Kyle Hayes

**Affiliations:** Mallinckrodt Pharmaceuticals, Bridgewater, New Jersey, USA.

**Keywords:** Acthar gel, CD4, CD8, repository corticotropin injection, RCI, T cells

## Abstract

Several inflammatory diseases are characterized by elevated T cell counts and high pro-inflammatory cytokine levels. Inhibiting T cell activity may reduce tissue damage associated with these diseases. Acthar^®^ Gel has potent anti-inflammatory properties, yet little is known about its effect on T cells. This study compared the effects of Acthar, synthetic adrenocorticotropic hormone 1–24 (ACTH_1–24_) depot, and prednisolone in a murine model of T cell activation. Assessments of CD4^+^ helper and CD8^+^ cytotoxic T cells and plasma concentrations of interferon-γ (IFN-γ), interleukin-2 (IL-2), and tumor necrosis factor-α (TNF-α) were made following anti-CD3-activation. Acthar significantly reduced the number of activated CD4^+^ and CD8^+^ T cells at amounts comparable to synthetic ACTH_1–24_ depot or prednisolone. However, Acthar reduced production of IFN-γ, IL-2, and TNF-α significantly more than the other drugs, suggesting that the *in vivo* immunomodulatory effects of Acthar on T cells are distinct from synthetic ACTH_1–24_ depot or prednisolone.

**Figure:**
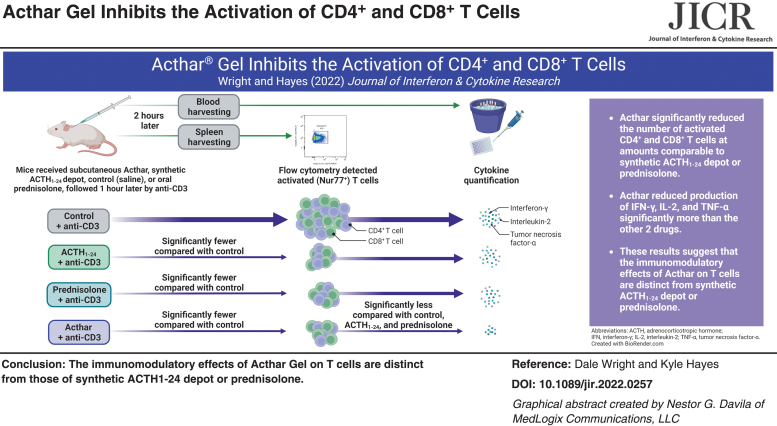


## Introduction

T cells are implicated in mediating many aspects of autoimmune inflammation. Their activity contributes to the pathophysiology of inflammatory diseases, including sarcoidosis, multiple sclerosis (MS), and rheumatoid arthritis (RA) (Kolios et al., 2021). Patients with these diseases often present with elevated counts of CD4^+^ helper T cells and CD8^+^ cytotoxic T cells and high levels of pro-inflammatory cytokines (Kolios et al., 2021; Wan and Flavell, 2009).

CD4^+^ T cells play critical roles in mediating adaptive immunity and help drive the destructive inflammatory immune response in autoimmune disorders (Wan and Flavell, 2009; Zhu et al., 2010). In patients with active sarcoidosis, CD4^+^ T cells accumulate in granulomatous lung tissue. These cells secrete pro-inflammatory cytokines, including interferon-γ (IFN-γ), interleukin-2 (IL-2), and tumor necrosis factor-α (TNF-α), which mediate local granuloma development (Broos et al., 2013; Oswald-Richter et al., 2013).

MS severity correlates with the number of active CD4^+^ T cells found in acute lesions, and IFN-γ, IL-2, and TNF-α are elevated in the blood of patients with MS (Bai et al., 2019; Chitnis, 2007; Peeters et al., 2017). RA is driven by abnormally activated CD4^+^ T cells hypersecreting IFN-γ and TNF-α, which continuously stimulate macrophages and osteoclasts and results in bone and cartilage degradation (Jiang et al., 2021; Skapenko et al., 2005).

Cytotoxic CD8^+^ T cells defend against intracellular pathogens and are integral in tumor surveillance (Skapenko et al., 2005). Mounting evidence suggests that CD8^+^ T cells help initiate, progress, and regulate pathogenic autoimmune responses (Skapenko et al., 2005). CD8^+^ T cell counts often are elevated in inflammatory disease and can produce high levels of TNF-α and IFN-γ, which may lead to the destruction of healthy tissue (Collier et al., 2021; Skapenko et al., 2005; Wan and Flavell, 2009).

In patients with RA, active CD8^+^ T cells accumulate in blood and synovial fluid and contribute to sustained inflammation by increasing pro-inflammatory cytokine secretion (Skapenko et al., 2005). Active CD8^+^ T cells that secrete TNF-α and IFN-γ are also concentrated in chronic MS lesions (Chitnis, 2007; Stojic-Vukanic et al., 2020; Zang et al., 2004). T cells offer a tractable target to reprogram the immune system and shift the balance toward regulation and homeostasis, rather than inappropriate activation and inflammation. Inhibiting inflammatory T cells can directly reduce tissue damage in autoimmune-mediated diseases (Pugliese, 2017).

Glucocorticoids and other therapeutics that block co-stimulatory pathways or target cytokine signaling [eg, Janus kinase/signal transducers and activators of transcription (JAK/STAT)] are known to inhibit T cell activation (Tanaka et al., 2022). The immunosuppressive capacity of glucocorticoids can inhibit T cell development, differentiation, and activity (Taves and Ashwell, 2021).

Glucocorticoids are integral in the standard of care for inflammatory diseases (Fleischmann et al., 2020; Mirsaeidi and Baughman, 2022; Perry et al., 2014); however, many patients experience refractory or relapsing-remitting disease that inadequately responds to standard of care anti-inflammatory therapies, including glucocorticoids (Askanase et al., 2020; Baughman et al., 2017; Fleischmann et al., 2020). Further, some patients cannot tolerate the short-term and long-term side effects of glucocorticoids. Thus, an unmet need exists for alternative treatments for these patients.

Acthar^®^ Gel (repository corticotropin injection) is a complex mixture of adrenocorticotropic hormone (ACTH) analogs and other pituitary peptides that is approved by the US Food and Drug Administration (FDA) to treat several inflammatory and autoimmune diseases involving activated CD4^+^ and CD8^+^ cells, including RA, MS, and sarcoidosis (Mallinckrodt Pharmaceuticals, 2021). Acthar binds and activates all 5 melanocortin receptor (MCR) subtypes, with its lowest full agonistic activity at melanocortin receptor 2 (MC2R), whereas synthetic ACTH_1–24_ depot shows its highest agonistic activity at MC2R (Huang et al., 2021).

The anti-inflammatory mechanism of Acthar was originally thought to be through stimulation of endogenous cortisol production via activation of MC2R on adrenocortical cells (Huang et al., 2021). However, Acthar induces less steroidogenesis than ACTH_1–24_ depot in both rats and humans, only slightly higher than normal endogenous levels (Huang et al., 2021; Poola et al., 2022; Wang et al., 2021). Further, Athar has non-steroidogenic immunomodulatory effects.

Clinical studies have demonstrated the efficacy of Acthar in patients with inflammatory diseases that are refractory to glucocorticoid treatment (Askanase et al., 2020; Baughman et al., 2017; Fleischmann et al., 2020; Wynn et al., 2022). Acthar also can directly inhibit B cell proliferation and antibody production (Olsen et al., 2015). The impact of Acthar on activated T cells has not been fully explored, but T cells express MC1R, MC3R, and MC5R, indicating that MCR agonists could directly affect T cell activation (Andersen et al., 2005; Arnason et al., 2013; Catania et al., 2010; Gong, 2011; Neumann Andersen et al., 2001; Wang et al., 2019).

Our objective was to assess whether Acthar inhibits the activity of CD4^+^ and CD8^+^ T cells and to compare those effects with the anti-inflammatory drugs synthetic ACTH_1–24_ depot and prednisolone. Nur77 was used as a marker of T cell activation, as it is rapidly upregulated upon stimulation of the T cell receptor/CD3 complex and is a more specific reporter of T cell activation than the more frequently used CD69 activation marker (Ashouri and Weiss, 2017). Common pro-inflammatory cytokines IFN-γ, IL-2, and TNF-α were also assessed to determine T cell activity.

## Methods

### Animal use and treatment

This study was performed in accordance with the National Institutes of Health's Guide for the Care and Use of Laboratory Animals (National Research Council, 2011). The study protocol was reviewed and approved by the Institutional Animal Care and Use Committee of Mallinckrodt Pharmaceuticals. Adult female C57BL/6 mice were obtained from Charles River Laboratories (Wilmington, MA) and allowed 3 days to acclimate with *ad libitum* access to food and water.

Mice were given a subcutaneous injection of 10, 40, or 400 U/kg of Acthar (Mallinckrodt Pharmaceuticals, Bridgewater, NJ, USA), 0.6 or 1.2 mg/kg of synthetic ACTH_1–24_ depot (Bachem Americas, Inc., Torrence, CA, USA), or saline (control), or a 5-mg/kg oral dose of prednisolone (Sigma-Aldrich, St. Louis, MO, USA). These doses were selected due to their established activity in rodent studies (Fiorucci et al., 2002; Huang et al., 2021; Lord et al., 2022). One hour later, mice received an intraperitoneal injection of 10 μg human anti-mouse anti-CD3 monoclonal antibody (HαM CD3 MAb; Clone#145-2C11; BioLegend) in saline to activate T cells. Blood and spleens were collected 2 h post anti-CD3 injection.

### Cell isolation and staining

Spleen cells were harvested, and red blood cells were removed using lysis buffer (Sigma-Aldrich). Cells were plated and washed twice in phosphate-buffered saline (PBS), and 25 μL mouse Fc block was added at 1:100 dilution for 15 min. Surface stains (HαM anti-CD4-FITC MAb or HαM anti-CD8-PEC7 MAb; 1:100, eBioscience) or viability stain (1:3,000; fixable violet) were added, and samples were incubated for 30 min and then washed with cold fluorescence-activated cell sorting (FACS) buffer (PBS, 2% fetal bovine serum, and 0.01% sodium azide).

Cells were incubated for 30 min in fixation/permeabilization solution (200 μL; Sigma-Aldrich), and they were then washed twice with permeabilization buffer. Samples were Fc blocked, then the recommended amount of Nur77 MAb PE (eBioscience) or isotype control (final dilution 1:100) was added. Samples were stained overnight at 2°C–8°C in the dark, washed twice, and resuspended in 200 μL FACS buffer.

A fluorescence minus one control was performed with anti-Nur77-PE MAb omitted from the staining protocol to determine the threshold fluorescence between background and positively labeled cells to be analyzed in Nur77^+^ expression analysis.

### Flow cytometry

The Attune flow cytometer (Thermo-Fisher Scientific, Inc.) was used to evaluate samples. The flow cytometry gating strategy included analysis of side scatter sorting for single cells, forward scatter sorting for lymphocytes, and then fixable violet sorting for live cells.

Nur77 expression was used as a specific reporter of T cell activation (Ashouri and Weiss, 2017); thus, cells were gated further by bidirectional sorting for CD4 and Nur77 expression or CD8 and Nur77 expression. Co-positive CD4^+^Nur77^+^ or CD8^+^Nur77^+^ cells were considered active.

### Pro-inflammatory cytokine analysis

Two hours after administration of anti-CD3, whole blood was collected by submandibular puncture. Plasma was collected and diluted 1:2 for IFN-γ, TNF-α, and IL-2 assessments using a chemiluminescence-based V-PLEX mouse kit assay (Meso Scale Discovery).

### Statistical analysis

One-way analysis of variance (ANOVA) with a Holm-Sidak multiple comparisons test was used to determine statistically significant differences in mean values. Outliers were excluded from ANOVA analysis if they were considered anti-CD3 stimulation failures (ie, if there was <10% increase in Nur77^+^ labeled cells or in cytokine levels compared with control after anti-CD3 administration).

## Results

**FIG. 1.**
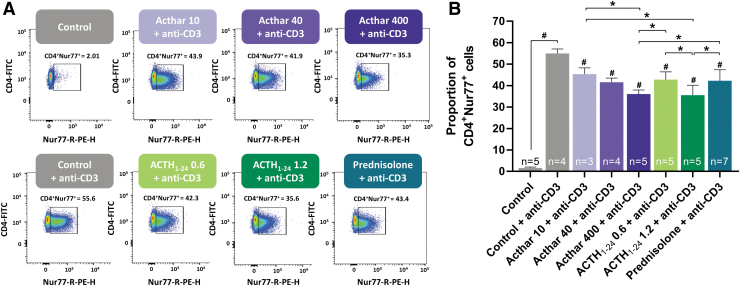
Flow cytometry analysis **(A)** and quantification **(B)** of anti-CD3-activated CD4^+^Nur77^+^ cells showing similar decreases in the number of active helper T cells with Acthar, synthetic ACTH_1–24_ depot, or prednisolone. Error bars represent standard deviation from the mean. Drug doses were as follows: Acthar (10, 40, or 100 U/kg), synthetic ACTH_1–24_ depot (0.6 or 1.2 mg/kg), prednisolone (5 mg/kg). ^#^*P* < 0.05 compared with control + anti-CD3, *P < 0.05. ACTH, adrenocorticotropic hormone.

**FIG. 2.**
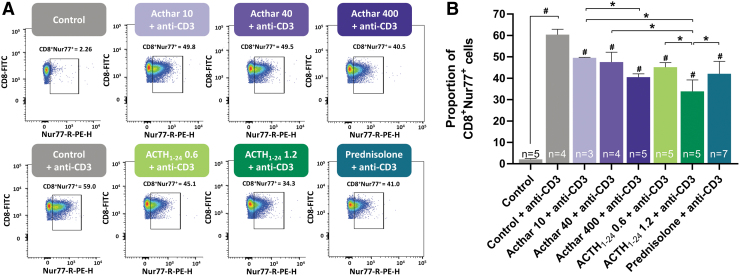
Flow cytometry analysis **(A)** and quantification **(B)** of anti-CD3-activated CD8^+^Nur77^+^ cells showing similar reductions in the number of active cytotoxic T cells with Acthar, synthetic ACTH_1–24_ depot, or prednisolone. Error bars represent standard deviation from the mean. Drug doses were as follows: Acthar (10, 40, or 100 U/kg), synthetic ACTH_1–24_ depot (0.6 or 1.2 mg/kg), prednisolone (5 mg/kg). ^#^*P* < 0.05 compared with control + anti-CD3, *P < 0.05.

Anti-CD3 administration significantly increased the proportion of CD4^+^Nur77^+^ cells (Fig. 1) and CD8^+^Nur77^+^ cells (Fig. 2). Compared with control, injection of any of the 3 anti-inflammatory drugs at all doses tested significantly reduced the proportion of anti-CD3-activated CD4^+^Nur77^+^ and CD8^+^Nur77^+^ cells. This reduction was dose-dependent, as the high doses of Acthar and synthetic ACTH_1–24_ depot reduced the mean proportion of CD4^+^Nur77^+^ and CD8^+^Nur77^+^ more than the low doses.

High doses of Acthar and synthetic ACTH_1–24_ depot similarly reduced the proportions of CD4^+^Nur77^+^ cells and significantly more than prednisolone. As for CD8^+^Nur77^+^ cells, the highest dose of synthetic ACTH_1–24_ depot reduced the proportion significantly more than prednisolone, but not significantly more than the highest dose of Acthar.

**FIG. 3.**
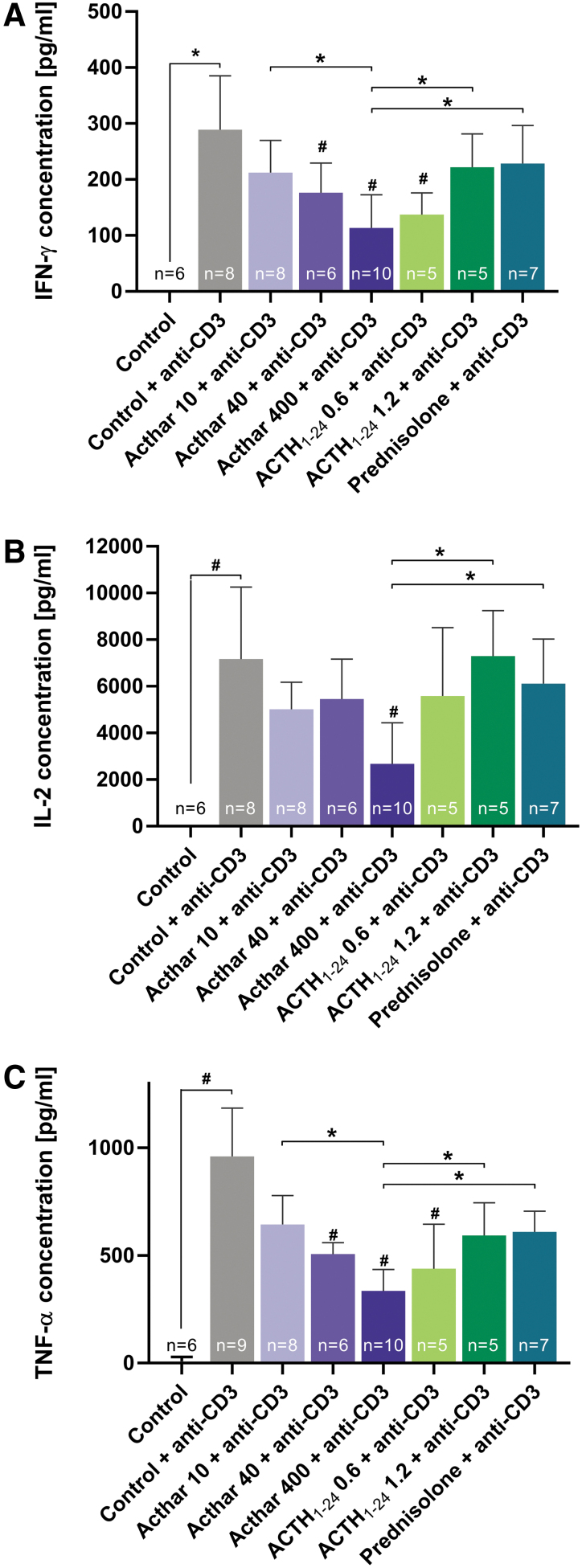
Cytokine analysis showing that high-dose Acthar significantly decreased plasma concentrations of IFN-γ **(A)**, IL-2 **(B)**, and TNF-α **(C)** more than high-dose synthetic ACTH_1–24_ depot or prednisolone. Error bars represent standard deviation from the mean. Drug doses were as follows: Acthar (10, 40, or 100 U/kg), synthetic ACTH_1–24_ depot (0.6 or 1.2 mg/kg), prednisolone (5 mg/kg). ^#^*P* < 0.05 compared with control + anti-CD3, *P < 0.05. IFN-γ, interferon-γ; IL-2, interleukin-2; TNF-α, tumor-necrosis factor-α.

Compared with control, anti-CD3 stimulation significantly increased IFN-γ, IL-2, and TNF-α plasma concentrations (Fig. 3). Administration of the highest dose of Acthar significantly reduced the anti-CD3-induced increases of all 3 cytokines. Only the lowest dose of synthetic ACTH_1–24_ depot significantly reduced IFN-γ and TNF-α levels, whereas prednisolone did not significantly affect any of the cytokine concentrations. Treatment with the highest dose of Acthar significantly reduced IFN-γ, IL-2, and TNF-α more than prednisolone or the highest dose of synthetic ACTH_1–24_ depot.

## Discussion

Given that activated CD4^+^ and CD8^+^ T cells contribute to the pathophysiology of inflammatory diseases including MS, RA, sarcoidosis, and autoimmune uveitis (Kolios et al., 2021), inhibition of T cell activity may lead to improved outcomes in patients with these diseases (Chitnis, 2007; Oswald-Richter et al., 2013; Peeters et al., 2017). Reduction of active CD4^+^ T cells in lung tissue and normalization of IL-2 levels are associated with spontaneous clinical resolution of sarcoidosis (Oswald-Richter et al., 2013). In patients with MS, the number of active CD4^+^ T cells correlates with disease severity; thus, inhibition of these cells may reduce disease signs, symptoms, and progression (Chitnis, 2007; Peeters et al., 2017). Acthar is FDA-approved to treat these and other inflammatory diseases.

Because synthetic ACTH_1–24_ depot has its strongest agonistic activity at MC2R (Huang et al., 2021) and its effects on cytokine concentrations did not differ significantly from those of prednisolone, the effects of ACTH_1–24_ depot were likely mediated by its steroidogenic mechanism. ACTH_1–24_ depot could directly affect T cells in a similar manner as Acthar, but the degree of effect may differ. Low-dose ACTH_1–24_ depot demonstrated greater reductions of cytokines than the high dose, which showed similar levels to those observed after treatment with prednisolone. This finding suggests that high doses may stimulate more steroidogenesis via MC2R, but low doses may preferentially activate other MCRs on T cells. Acthar induces much less endogenous glucocorticoid release than synthetic ACTH_1–24_ depot (Huang et al., 2021; Poola et al., 2022), yet Acthar reduced the amount of CD4^+^Nur77^+^ and CD8^+^Nur77^+^ T cells and proinflammatory cytokines at levels comparable to or greater than synthetic ACTH_1–24_ depot or prednisolone.

Further, only Acthar significantly inhibited IL-2, a cytokine that is correlated with increased severity of inflammatory diseases including MS and RA (Kondo et al., 2021; Peeters et al., 2017). Acthar has also demonstrated a greater reduction of B cell activation, proliferation, and immunoglobulin production compared with ACTH_1–24_ depot (Benko et al., 2021). Taken together, these results suggest a unique mechanism of action for Acthar that differs from synthetic ACTH_1–24_ depot or prednisolone. The effects of Acthar on T cells may be primarily due to its steroid-independent mechanism of direct immunomodulatory activation of MCRs on T cells.

These results support that the anti-inflammatory effects of Acthar are partially due to the reduction of activated CD4^+^ and CD8^+^ T cells and their release of proinflammatory cytokines beyond steroidogenesis. Acthar promotes substantially lower steroidogenesis compared with synthetic ACTH_1–24_ depot (Huang et al., 2021; Poola et al., 2022). Future studies are warranted to determine which MCR subtypes and signaling mechanisms mediate the unique effects of Acthar reported here.

## Conclusions

Acthar significantly reduced the number of anti-CD3-activated CD4^+^ and CD8^+^ T cells *in vivo* at levels comparable to synthetic ACTH_1–24_ depot or prednisolone. However, Acthar reduced T cell-mediated production of the pro-inflammatory cytokines IFN-γ, IL-2, and TNF-α significantly more than the other 2 drugs. These results suggest that Acthar has a distinct mechanism of action from synthetic ACTH_1–24_ depot or prednisolone in reducing T cell activity and support that immunomodulation of T cells may contribute to the anti-inflammatory effects of Acthar.

Thus, Acthar may be beneficial for the treatment of inflammatory diseases characterized by active CD4^+^ and CD8^+^ T cells, particularly in patients who do not respond adequately to standard of care therapies, such as glucocorticoids.
